# Comparison of clinical outcomes of using the nonirradiated and irradiated allograft for anterior cruciate ligament (ACL) reconstruction: A systematic review update and meta-analysis

**DOI:** 10.1097/MD.0000000000029990

**Published:** 2022-08-12

**Authors:** Yan Liu, Xuegang Liu, Yancai Liu, Shan Yang

**Affiliations:** a Outpatient Department of Hebei Armed Police Corps Hospital, Shijiazhuang, Hebei, P.R. China; b Department of General Surgery, The Fourth People’s Hospital of Hengshui, Hengshui, Hebei, P.R. China; c Department of Pathology, The Fourth People’s Hospital of Hengshui, Hengshui, Hebei, P.R. China; d Department of Pain, the Third Hospital of Hebei Medical University. Shijiazhuang, Hebei, P.R. China.

**Keywords:** ACL, irradiated allograft, meta analysis

## Abstract

**Background:**

This study was a systematic review comparing the clinical outcomes of using the nonirradiated and irradiated allograft for anterior cruciate ligament (ACL) reconstruction.

**Methods:**

A comprehensive literature search was conducted using multiple databases, including Medline, Embase, and Cochrane. All databases were searched from the earliest records through August 2019 using the following Boolean operators: irradiated AND nonirradiated AND ACL AND allograft. All prospective and retrospective controlled trials were retrieved that directly compared physical examination and knee function scores and patient-rated outcomes between the nonirradiated and irradiated allograft for ACL reconstruction.

**Results:**

Three prospective and 2 retrospective articles were identified by the search, and the findings suggested that the nonirradiated allografts were superior to the irradiated allografts based on improved knee joint functional scores and decreased failure rate, even though there was no significantly difference with respect to overall IKDC, range of motion, vertical jump test, and one-leg hop test.

**Conclusions:**

Irradiated allograft should be limited to be used in ACL surgery and further research into new alternative sterilization techniques are needed to avoiding the disease transmission without interference with the biomechanical properties of the grafts.

## 1. Introduction

Rupture of the anterior cruciate ligament (ACL) is a common sports injury. Arthroscopically assisted ACL reconstruction is the most common management. Autograft is routinely used, such as hamstring tendon autografts and BPTB.^[1,2]^ However, there are potential complications with the use of autograft, including increased operative time, small tendon size, additional scars, nerve damage to the saphenous nerve.^[3,4]^ In the past decade, the use of allograft tissue in ACL reconstruction has risen tremendously. There are many advantages of using allografts for reconstruction, including no donor-site morbidity, shorter operating time, less pain, easier rehabilitation, and smaller surgical incisions.^[5,6]^

However, a potential disadvantage in the use of allograft tissue is the disease transmission, both bacterial and viral, such as HIV, hepatitis B, and so on. To minimize the risk of disease transmission, gamma irradiation was used to sterilize allografts. Although many studies suggest that gamma irradiation decreases the initial biomechanical properties of allograft in a dose-dependent manner.^[7,8]^ This makes surgeons more difficult to choose an ideal graft for patients. However, whether the alteration in biomechanical property affects the clinical outcomes of ACL reconstruction with irradiated allograft remains controversial.

The purpose of this up-to-date meta-analysis was to directly compare the clinical outcomes of ACL reconstruction with the nonirradiated and irradiated allograft to identify the most appropriate allograft for the reconstruction of ACL.

## 2. Materials and Methods

### 2.1. Literature search

Three independent investigators performed an electronic search of the following databases: PubMed Medline, Embase, and the Cochrane Registry of Clinical Trials. All databases were searched from the earliest records through August 2019. We strictly followed the methods established in the Cochrane Handbook for Systematic Reviews of Interventions 5.0.2, and the Preferred Reporting Items for Systematic Reviews and Meta-Analyses 2009 checklist.^[9]^ The search used the following terms and Boolean operators: irradiated AND nonirradiated AND anterior cruciate ligament AND allograft. We applied no restrictions on language or year of publication. Additional relevant studies were identified by perusing the references of retrieved studies and review articles.

### 2.2. Inclusion and exclusion criteria

The criteria for inclusion of the studies included: (1) controlled clinical trials; (2) studies that directly compared the irradiated and nonirradiated allograft, with available clinical outcomes. Exclusion criteria were (1) studies where no comparative data were provided, or (2) cadaver studies.

### 2.3. Data extraction and outcome measures

Once the studies met the inclusion criteria, data were extracted by 3 reviewers independently. For each trial, data were collected on the following characteristics: *patient demographics*, *Overall IKDC*, *Range of motion*, *Vertical jump test*, *One-leg hop test*, *Pivot shift test*, *ADT*, *Failure cases*, *Lachman test*, *KT-2000 Side-to-side differences*, *Subjective IKDC*, *Cincinnati knee score*, *Lysholm score*, *Tegner score.*

### 2.4. Quality assessment

The methodological quality of the included trials was evaluated independently by the reviewers using a specific tool for assessing risk of bias, as recommended by the Cochrane Collaboration to assess methodological quality of clinical trials. This comprises a description and judgment for each entry in a “risk of bias” table, where each entry addresses a specific feature of the study. The judgment for each entry involved answering a question, with answers “yes” indicating a low risk of bias, “no” indicating a high risk of bias, and “unclear” indicating either a lack of information or uncertainty of the potential for bias.^[10]^

### 2.5. Statistical analysis

Statistical analyses were conducted using the software Review Manager 4.2, which was provided by Cochrane Collaboration. The treatment effects were expressed as risk ratios (RR), with 95% confidence intervals (CI) for dichotomous outcomes and mean differences (MD) with 95% CI for continuous outcomes. Heterogeneity was tested using the Chi-square test with significance set at *P* < .1. The I-square test was also used to quantify the effect of heterogeneity with an I^2^ of 50% or higher representing substantial heterogeneity. If there was no statistical evidence of heterogeneity, a fixed-effects model was used; otherwise, a random-effects model was adopted. If standard deviation was required to be calculated from raw data, SPSS 13.0 software was used.

## 3. Results

**Figure 1.**
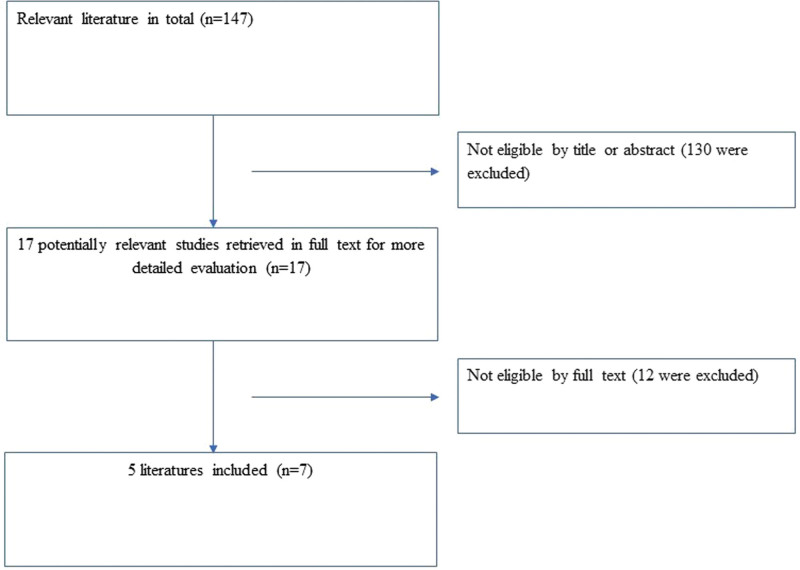
A PRISMA flow chart illustrated the selection of studies included in our systematic review.

A flow chart of the study selection process is presented in Figure 1. An initial search identified 143 articles using the search protocol. After further evaluation of the titles, text words, and abstracts, 17 potentially relevant studies were selected for full-text examination. Finally, 5 published articles^[11–15]^ were determined as appropriate for inclusion in the study. A funnel plot analysis was unable to be performed because of insufficient studies identified, as both visual examination and statistical analysis of funnel plots have limited power to detect bias if the number of trials is small.

### 3.1. Characteristics and interventions

The demographic characteristics of the patients in the 7 studies are presented in Table 1. Three prospective studies^[11,12,15]^ and 2 retrospective comparative studies^[13,14]^ were retrieved. A total of 374 patients were included: 191 in the nonirradiated group and 183 in the irradiated group. Study characteristics were described in all 5 studies (Table 2). Summary of the clinical outcomes comparing nonirradiated and irradiated is presented in Table 3.

**Table 1:** Characteristics and methodological quality of the included studies.

No.	Author and publication date	Adequate sequence generation?*	Allocation concealment †	Blinding?‡	Incomplete outcome data addressed?§	Free of selective reporting?‖	Free of other bias?¶
1	Tian et al (2017)^[15]^	Yes	Yes	Yes	Yes	Yes	Yes
2	Sun et al (2012)^[11]^	Yes	Yes	Yes	Yes	Yes	Yes
3	Sun et al (2009)^[12]^	Yes	Yes	Yes	Yes	Yes	Yes
4	Rappe et al (2007)^[14]^	Unclear	Unclear	Unclear	Yes	Yes	Yes
5	Guo 2012^[13]^	Unclear	Unclear	Unclear	yes	Yes	Yes

**Table 2:** Study characteristics.

No.	Author and publication date	Study design	Sample size	Management		Mean age (yrs)		Minimum length of follow-up
				Nonirradiated	Irradiated	Nonirradiated	Irradiated	
1	Tian et al (2017)^[15]^	Prospective study	83	44	39	30.2	29.8	Nonirradiated 5.8 yrs
								Irradiated 5.6 yrs
2	Sun et al (2012)^[11]^	Prospective study	69	38	31	31.7	30.3	Nonirradiated 42.1 mos
								Irradiated 43 mos
3	Sun et al (2009)^[12]^	Prospective study	66	34	32	31.8	30.1	Nonirradiated 27.3 mos
								Irradiated 25.6 mos
4	Rappe et al (2007)^[14]^	Retrospective study	75	42	33	27	26	6 mos
5	Guo 2012^[13]^	Retrospective study	81	33	68	25.3	25.7	6.7 yrs

**Table 3:** Summary of clinical outcomes comparing trans-tibial and outside to inside drilling techniques.

No.	Author and publication date		Failure cases	Overall IKDC (normal and nearly normal)	Range of motion (normaland nearly normal)	Vertical jump test (normal and nearly normal)	One-leg hop test (normaland nearly normal)	Pivot shift test (0&I)	ADT (0&I)	Lachman test (0&I)	KT-2000 Side-to-side differences	Subjective IKDC	Cincinnati knee score	Lysholm score	Tegner score
1	Tian et al (2017)^[15]^	Nonirradiated	0	40	42	42	42	44.	42	41	2.5 ± 0.8	88.2 ± 10.3	89.8 ± 11.2	88.8 ± 11.3	7.7 ± 1.0
		Irradiated	0	35	37.	36	36	35	29	28	5.5 ± 1.1	84.3 ± 11.1	86.2 ± 10.4	85.9 ± 9.3	7.4 ± 1.1
2	Sun et al (2012)^[11]^	Nonirradiated	4	34	36	35	36	38	35	34	2.7 ± 0.8	88 ± 9	90 ± 9	90 ± 7	7.3
		Irradiated	10	27	29	27	28	27	22	21	5.6 ± 3.1	83 ± 10	85 ± 13	87 ± 11	7.1
3	Sun et al (2009)^[12]^	Nonirradiated	3	31	32	32	33	34	32	31	2.6 ± 0.9	89 ± 9	91 ± 12	91 ± 8	7.5 ± 1.5
		Irradiated	11	28	30	28	29	28	23	21	5.5 ± 3.6	84 ± 12	85 ± 14	87 ± 10	7.0 ± 1.7
4	Rappe et al (2007)^[14]^	Nonirradiated	1	–	–	–	–	–	–	–	–	–	–	–	–
		Irradiated	11	–	–	–	–	–	–	–	–	–	–	–	–
5	Guo 2012^[13]^	Nonirradiated	0	–	–	–	–	30	–	–	2.6 ± 0.4	–	–	85.6 ± 10.1	4.1 ± 0.8
		Irradiated	6	–	–	–	–	55	–	–	4.1 ± 0.6	–	–	79.8 ± 8.5	3.7 ± 0.6

### 3.2. Meta-analysis of clinical results

#### 3.2.1. Overall IKDC.

**Figure 2.**
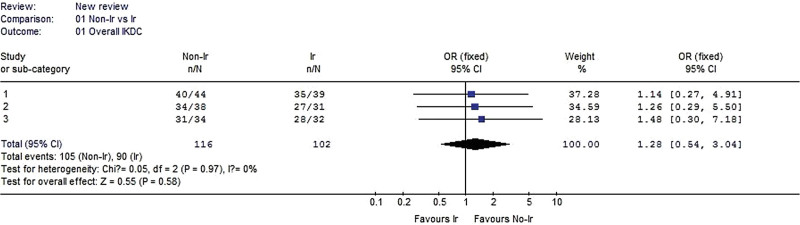
Forest plot of Overall IKDC between the nonirradiated and irradiated allograft using for ACL reconstruction. ACL = anterior cruciate ligament, IKDC = International Knee Documentation Committee.

Three studies reported overall IKDC data. There was no significant difference between the irradiated and nonirradiated groups (χ^2^ = 0.05, *P* = .97, I^2^ = 0%). There was no significant difference (OR 1.28, 95% CI: 0.54–3.04, *P* = .58, Fig. 2).

#### 3.2.2. Range of motion.

**Figure 3.**
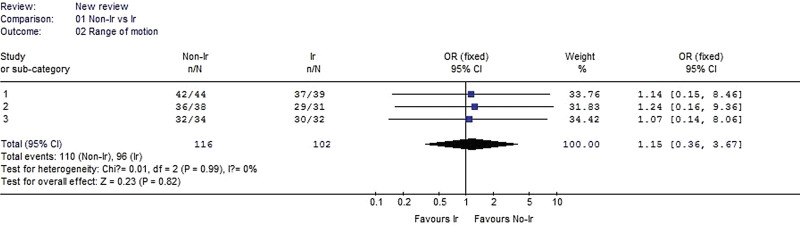
Forest plot of range of motion between the nonirradiated and irradiated allograft using for ACL reconstruction. ACL = anterior cruciate ligament.

Three studies reported range of motion and were included in the analysis. No heterogeneity was detected when the data from the 3 studies were pooled (χ^2^ = 0.01, *P* = .99, I^2^ = 0%). No significant difference in range of motion between the irradiated and nonirradiated groups was noted. (OR 1.15, 95% CI: 0.36–3.67, *P* = .82, Fig. 3).

#### 3.2.3. Vertical jump test.

**Figure 4.**
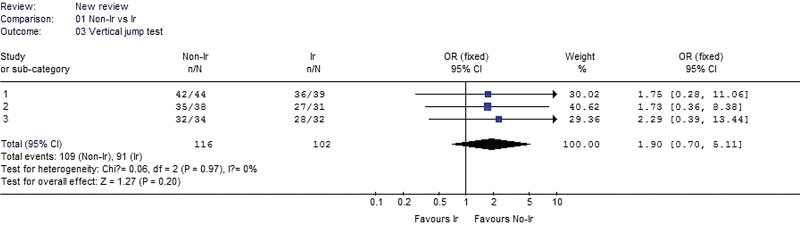
Forest plot of vertical jump test between the nonirradiated and irradiated allograft using for ACL reconstruction. ACL = anterior cruciate ligament.

Three studies reported vertical jump test data. No heterogeneity was detected when the data from the 3 studies were pooled (χ^2^ = 0.06, *P* = .97, I^2^ = 0%). The pooled data of vertical jump test indicated no significant difference between the irradiated and nonirradiated groups (OR 1.90, 95% CI: 0.70–5.11, *P* = .20, Fig. 4).

#### 3.2.4. One-leg hop test.

**Figure 5.**
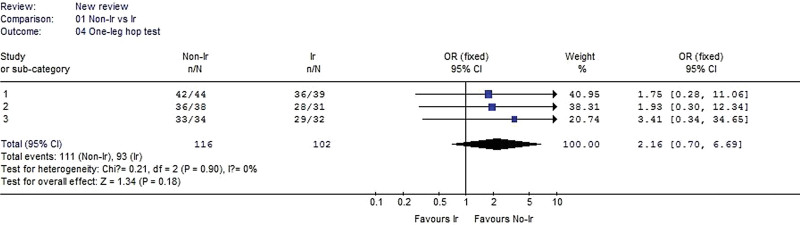
Forest plot of One-leg hop test between the nonirradiated and irradiated allograft using for ACL reconstruction. ACL = anterior cruciate ligament.

Three studies reported one-leg hop test data. No heterogeneity was detected when the data from the 3 studies were pooled (χ^2^ = 0.21, *P* = .90, I^2^ = 0%). The pooled data of one-leg hop test indicated no significant difference between the irradiated and nonirradiated groups (OR 2.16, 95% CI: 0.70–6.69, *P* = .18, Fig. 5).

#### 3.2.5. Pivot shift test.

**Figure 6.**
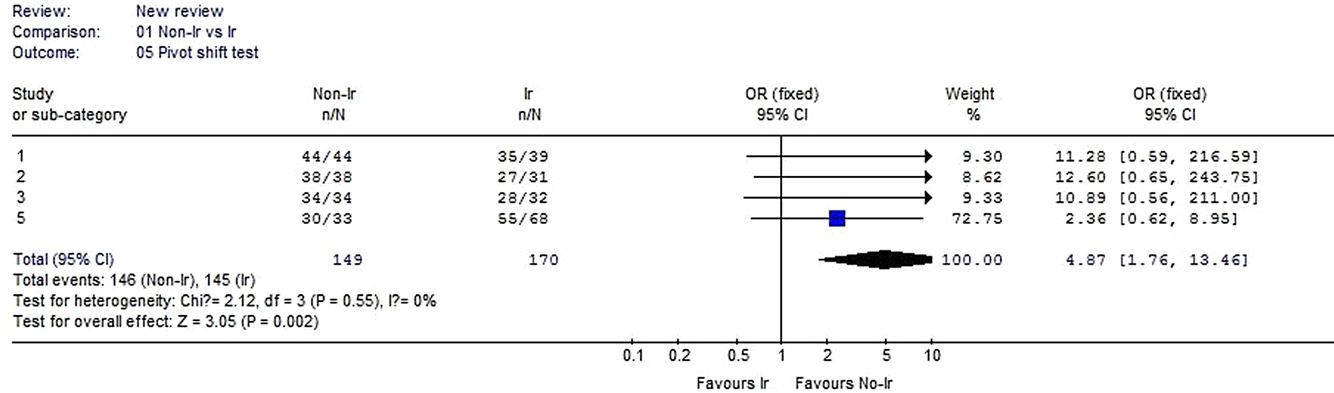
Forest plot of Pivot shift test between the nonirradiated and irradiated allograft using for ACL reconstruction. ACL = anterior cruciate ligament.

Four studies reported the pivot shift test data. No heterogeneity was detected when the data from the 4 studies were pooled (χ^2^ = 2.12, *P* = .55, I^2^ = 0%). The result showed that pivot shift test was significantly better in the nonirradiated group than in the irradiated group for all 4 of these studies (OR 4.87, 95% CI: 1.76–13.46, *P* = .002, Fig. 6).

#### 3.2.6. Anterior drawer test.

**Figure 7.**
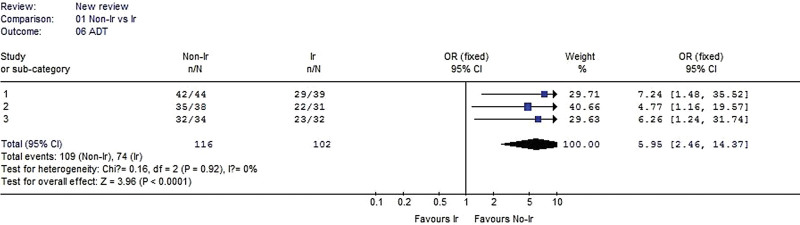
Forest plot of ADT between the nonirradiated and irradiated allograft using for ACL reconstruction. ACL = anterior cruciate ligament, ADT = anterior drawer test.

Three studies were included in the analysis. No heterogeneity was detected when the data from the 3 studies were pooled (χ^2^ = 0.16, *P* = .92, I^2^ = 0%). The result showed that ADT was significantly better in the nonirradiated group than in the irradiated group for all 3 of these studies (OR 5.59, 95% CI: 2.46–14.37, *P* < .0001, Fig. 7).

#### 3.2.7. Failure rate.

**Figure 8.**
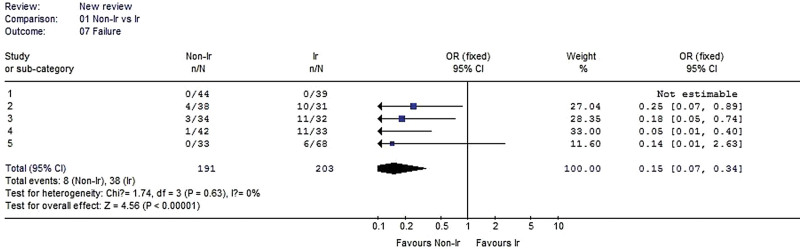
Forest plot of failure cases between the nonirradiated and irradiated allograft using for ACL reconstruction. ACL = anterior cruciate ligament.

Five studies were included in the analysis. No heterogeneity was detected when the data from the 3 studies were pooled (χ^2^ = 1.74, *P* = .63, I^2^ = 0%). The result showed that failure rate was significantly lower in the nonirradiated group than in the irradiated group (OR 0.15, 95% CI: 0.07–0.34, *P* < .00001, Fig. 8).

#### 3.2.8. Lachman test.

**Figure 9.**
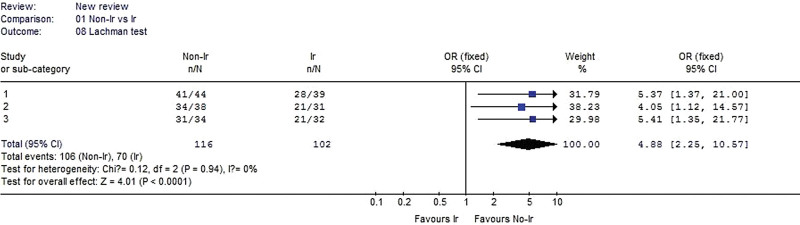
Forest plot of Lachman test between the nonirradiated and irradiated allograft using for ACL reconstruction. ACL = anterior cruciate ligament.

Three studies reported Lachman test data. No heterogeneity was detected when the data from the 3 studies were pooled (χ^2^ = 0.12, *P* = .94, I^2^ = 0%). The result showed that Lachman test was significantly better in the nonirradiated group than in the irradiated group (OR 4.88, 95% CI: 2.25–10.57, *P* < .0001, Fig. 9).

#### 3.2.9. KT-2000 side-to-side differences.

**Figure 10.**
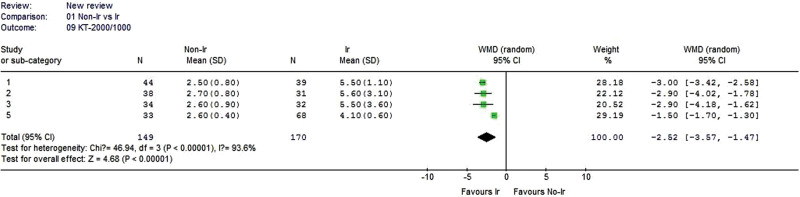
Forest plot of KT-2000 side-to-side differences between the nonirradiated and irradiated allograft using for ACL reconstruction. ACL = anterior cruciate ligament.

Four studies reported KT-2000/1000 side-to-side differences data. Significant heterogeneity was found when the data from the 4 studies were pooled (χ^2^ = 46.94, *P* < .00001, I^2^ = 93.6%), and a random-effects model was adopted. The nonirradiated group had a significantly lower KT-2000/1000 side-to-side differences than the irradiated group (WMD –2.52, 95% CI: –3.57 to –1.47, *P* < .00001, Fig. 10).

#### 3.2.10. Subjective IKDC.

**Figure 11.**
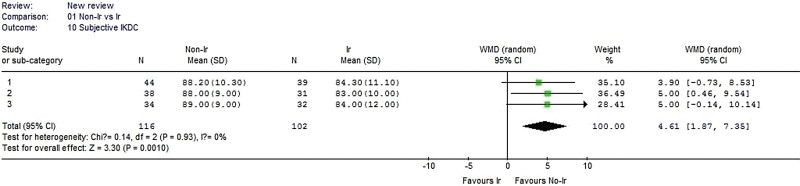
Forest plot of Subjective IKDC between the nonirradiated and irradiated allograft using for ACL reconstruction. ACL = anterior cruciate ligament, IKDC = International Knee Documentation Committee.

Three studies reported subjective IKDC data. No heterogeneity was detected when the data from the 3 studies were pooled (χ^2^ = 0.14, *P* = .93, I^2^ = 0%). The result showed that the nonirradiated group had a significantly greater subjective IKDC than the irradiated group (WMD 4.61, 95% CI: 1.87–7.35, *P* = .0010, Fig. 11).

#### 3.2.11. Cincinnati knee score.

**Figure 12.**
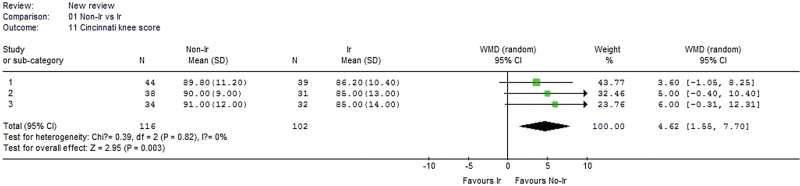
Forest plot of Cincinnati knee score between the nonirradiated and irradiated allograft using for ACL reconstruction. ACL = anterior cruciate ligament.

Three studies reported the Cincinnati knee score and were included in the analysis. No heterogeneity was detected when the data from the 3 studies were pooled (χ^2^ = 0.39, *P* = .82, I^2^ = 0%). The result showed that the nonirradiated group had a significantly better Cincinnati knee score than the irradiated group (WMD 4.62, 95% CI: 1.55–7.70, *P* = .003, Fig. 12).

#### 3.2.12. Lysholm score.

**Figure 13.**
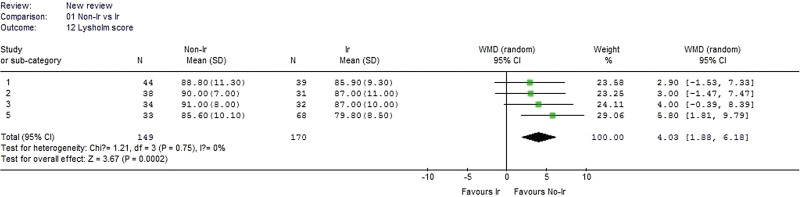
Forest plot of Lysholm score between the nonirradiated and irradiated allograft using for ACL reconstruction. ACL = anterior cruciate ligament.

Three studies reported the Lysholm score and were included in the analysis. No heterogeneity was detected when the data from the 3 studies were pooled (χ^2^ = 1.21, *P* = .75, I^2^ = 0%). The nonirradiated group had a significantly better Lysholm score than the irradiated group (WMD 4.03, 95% CI: 1.88–6.18, *P* = .0002, Fig. 13).

#### 3.2.13. Tegner score.

**Figure 14.**
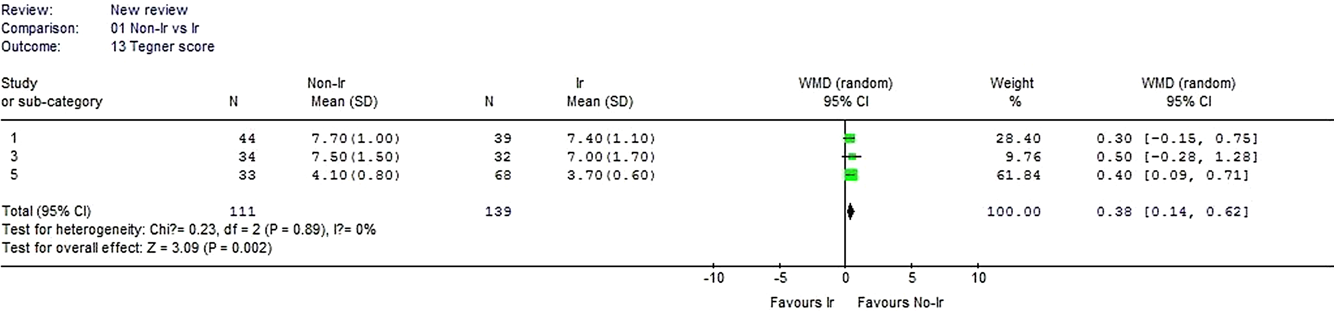
Forest plot of Tegner score between the nonirradiated and irradiated allograft using for ACL reconstruction. ACL = anterior cruciate ligament.

Three studies reported the Tegner score and were included in the analysis. No heterogeneity was detected when the data from the 3 studies were pooled (χ^2^ = 0.23, *P* = .89, I^2^ = 0%). The nonirradiated group had a significantly better Tegner score than the irradiated group (WMD 0.38, 95% CI: 0.14–0.62, *P* = .002, Fig. 14).

## 4. Discussion

This meta-analysis evaluated clinical function after the use of irradiated and nonirradiated allografts for ACL reconstruction. The most important finding of this study was that the nonirradiated allografts was superior to the irradiated allografts based on improved knee joint functional scores and decreased failure rate, even though there was no significantly difference with respect to Overall IKDC, Range of motion, Vertical jump test, and One-leg hop test.

As a common sports injury, rupture of the ACL need to be reconstructed in most times.^[16,17]^ Arthroscopically assisted ACL reconstruction with autografts is considered the most common management for a variety of reasons.^[18]^ However, surgeons have to face to many potential complications, including donor-site morbidity, increased operative time, small tendon size, additional scars, nerve damage to the saphenous nerve. What is more, due to the increased operative and anesthesia time, the cost may increase when compare with allograft ACL reconstruction.^[19]^ Thus, this makes surgeons choose allografts in an attempt to avoid such problems.

A systematic review compared autografts and allografts for ACL reconstruction, the results suggests no difference in rupture rates and clinical outcomes.^[20]^ While several studies also compared autograft and allograft used in ACL reconstruction, the results were various due to the variety of tissues used, different surgical technique and postoperative rehabilitation protocols.^[3,21–24]^ In a recent systematic review, Zeng et al^[25]^ reported that there were no significant differences between autograft and nonirradiated allograft regarding to function and stability, whereas both autograft and nonirradiated allograft are superiors than irradiated allograft, and the difference is significant.

One of the major concerns with the use of allograft is the risk of disease transmission.^[26]^ Gamma irradiation which has known bactericidal and virucidal properties is the most popular method of sterilizing tissue transplants.^[27]^ However, biomechanical research showed that allograft irradiation has adverse effects on biomechanical properties of allograft in a dose-dependent fashion.^[5,7]^ Fideler et al^[28]^ reported that the dose of 2.5 Mrad was just bacteriocidal, and doses of 3 to 4 Mrad were necessary to inactivate the virus, such as HIV. Whereas Curran et al^[5]^ demonstrated that doses as low as 2 Mrad resulted in a statistically significant reduction the initial stiffness and strength of tendon allograft. When irradiated grafts were used for ACL reconstruction, the alteration in biomechanical properties may affect the clinical outcomes and failure rate. Rappe et al^[14]^and Sun et al^[12]^reported similar results, a significant increased in the failure rate in the irradiated allograft group. Conversely, Rihn et al^[29]^ compared irradiated BPTB allograft and BPTB autograft, the result showed that no adverse effect of irradiation on clinical outcome.

In the present review study, most of the clinical functional scores were significant better in nonirradiated group, including, subjective IKDC (*P* = .0010), Cincinnati knee score (*P* = .003), Lysholm score (*P* = .0002), Tegner score (*P* = .002). That means patients feel more comfortable when using nonirradiated allograft for ACL reconstruction, even though there was no significant difference in Overall IKDC (*P* = .58). No significant differences were found between the 2 groups according to the range of motion (*P* = .82), vertical jump test (*P* = .20), and one-leg hop test (*P* = .18). But there was an increase in anterior laxity or rate of graft rupture in patients who underwent reconstruction with irradiated allograft according to *the ADT* (*P* < .0001), failure rate (*P <* .00001), Lachman test (*P <* .0001), and maximal manual KT-2000 test (*P <* .00001). The rate of rotational instability also increased according to pivot shift test (*P* = .002). Thus, we do not suggest surgeon to use irradiated allograft in ACL reconstruction. Many antibiotic soaks can limit bioburden and associated immune response, such as various chemical rinses (e.g., peracetic acid) and proprietary treatments (e.g., AlloWash [LifeNet Health, Virginia Beach, VA]). These methods are not without potential harmful side effects. Ethylene oxide has been largely eliminated from current chemical processing practices because of strong associations with intense foreign body reaction, chronic synovitis, and graft dissolution.^[30–32]^ Even so, further research into new alternative sterilization techniques are needed to avoiding the disease transmission without interference with the biomechanical properties of the grafts.

There are some limitations in the present study: (1) the number of trials included was not so adequate, which just had 5 separate trials; (2) we cannot include some unpublished studies and data; (3) the quality of some trials, which did not provide adequate randomization and blinding method, was not high enough. These limitations should be avoided as far as possible when drafting new trials.

## 5. Conclusion

The nonirradiated allografts were superior to the irradiated allografts based on improved knee joint functional scores and decreased failure rate, even though there was no significantly difference with respect to overall IKDC, range of motion, vertical jump test, and one-leg hop test. Irradiated allograft should be limited to be used in ACL surgery and further research into new alternative sterilization techniques are needed to avoiding the disease transmission without interference with the biomechanical properties of the grafts.

## Author contributions

Conceptualization: Yan Liu, Shan Yang.

Data curation: Xuegang Liu , Shan Yang.

Formal analysis: Yancai Liu, Shan Yang.

Writing – original draft: Zeming Liu, Shan Yang.

Writing – review & editing: Shan Yang.

## Footnotes

* Was the allocation sequence adequately generated?

† Was allocation adequately concealed?

‡ Was knowledge of the allocated interventions adequately prevented during the study?

§ Were incomplete outcome data adequately addressed?

‖ Are reports of the study free of suggestion of selective outcome reporting?

¶ Was the study apparently free of other problems that could put it at a risk of bias?
